# A Phase 1/2 study of the PD-L1 inhibitor, BGB-A333, alone and in combination with the PD-1 inhibitor, tislelizumab, in patients with advanced solid tumours

**DOI:** 10.1038/s41416-022-02128-3

**Published:** 2023-02-16

**Authors:** Jayesh Desai, Peter Fong, Victor Moreno, Sophia Frentzas, Tarek Meniawy, Ben Markman, Mark Voskoboynik, Tahmina Rahman, Nageshwar Budha, John Wu, Jin Marlow, Silu Yang, Emiliano Calvo, Juan Martin-Liberal

**Affiliations:** 1 Peter MacCallum Cancer Centre and Sir Peter MacCallum Department of Oncology, University of Melbourne, Melbourne, VIC Australia; 2 Royal Melbourne Hospital, Parkville, VIC Australia; 3 The University of Auckland and Auckland City Hospital, Auckland, New Zealand; 4 START Madrid-FJD, Hospital Fundación Jiménez Díaz, Madrid, Spain; 5 Monash Health, Clayton, VIC Australia; 6 Monash University, Clayton, VIC Australia; 7 Linear Clinical Research and University of Western Australia, Nedlands, WA Australia; 8 Alfred Hospital, Melbourne, VIC Australia; 9 BeiGene USA, Inc., San Mateo, CA USA; 10 BeiGene (Beijing) Co., Ltd., Beijing, China; 11 START Madrid-CIOCC, Centro Integral Oncológico Clara Campal, Madrid, Spain; 12 Catalan Institute of Oncology (ICO), Hospitalet, Barcelona, Spain

**Keywords:** Cancer immunotherapy, Targeted therapies

## Abstract

**Background:**

Many patients do not respond or eventually relapse on treatment with programmed cell death protein-1 (PD-1)/programmed death-ligand 1 (PD-L1) checkpoint inhibitors due to secondary or acquired resistance; therefore, there is a need to investigate novel PD-1/PD-L1 inhibitors.

**Methods:**

This open-label, non-randomised study investigated the safety and anti-tumour activity of BGB-A333, a PD-L1 inhibitor, alone and in combination with tislelizumab in patients with advanced solid tumours with progression during/after standard therapy. The primary objectives were to determine the recommended Phase 2 dose (RP2D), safety and tolerability for BGB-A333 alone and in combination with tislelizumab (Phase 1a/1b) and to determine the overall response rate (ORR) with BGB-A333 plus tislelizumab (Phase 2).

**Results:**

Overall, 39 patients across Phase 1a (*N* = 15), 1b (*N* = 12) and 2 (*N* = 12) were enroled. In Phase 1a, an RP2D of 1350 mg was determined. In Phase 1a and 1b/2, serious treatment-emergent adverse events (TEAEs) were reported in five and eight patients, respectively. Two patients experienced TEAEs that led to death. In Phase 2, the ORR was 41.7% (*n* = 5/12; 95% confidence interval: 15.17%, 72.33%).

**Conclusions:**

TEAEs reported with BGB-A333 were consistent with other PD-L1 inhibitors. Encouraging preliminary anti-tumour activity was observed with BGB-A333 in combination with tislelizumab.

**Clinical trial registration:**

NCT03379259.

## Background

Programmed cell death protein-1 (PD-1) and its ligand, programmed death-ligand 1 (PD-L1), are immune checkpoint proteins that play critical roles in the immune modulation of tumour progression in a wide variety of tumour types [1, 2], making them suitable targets for cancer immunotherapy. PD-1/PD-L1 inhibitor monotherapy has demonstrated efficacy in various solid tumour types, including mismatch repair-deficient/microsatellite instability-high tumours, high tumour mutation burden tumours and some PD-L1-high tumours [3–5]. Broadly, compared with conventional therapies, treatment with PD-1/PD-L1 inhibitor monotherapy has been associated with greater tumour response rates, and has been found to decrease the risk of death in both PD-L1-positive and PD-L1-negative patients, although efficacy is highly variable between tumour types [5]. PD-1/PD-L1 inhibitors have received US Food and Drug Administration (FDA) approval for many tumour types, most of which have not required PD-L1 expression as a predictive biomarker [4].

Although treatment with PD-1/PD-L1 inhibitor monotherapy provides improved responses, prolonged survival and fewer toxicities compared with conventional therapies in many tumour types, a significant proportion of patients do not respond or eventually relapse on treatment due to secondary or acquired resistance [2, 5–8]. Overall response rates (ORRs) for patients receiving monotherapy with established PD-1/PD-L1 inhibitors are typically less than 30%, and are often considerably beneath this figure, with a preponderance of partial rather than complete responses [5]. Therefore, there is scope for the development of novel PD-1/PD-L1 inhibitors.

BGB-A333 is an investigational humanised monoclonal antibody against PD-L1 that has demonstrated anti-tumour activity in xenograft models [9]. BGB-A333 blocks the interaction between PD-L1 and CD80 (B7-1), which in turn releases inhibitory signals to T cells, enhances T-cell expansion and prevents T-cell anergy induction [10]. Additionally, BGB-A333 exhibits no or very low binding to C1q on all Fc gamma receptors (FcγRs) in in vitro binding assays, suggesting low or no antibody-dependent cell-mediated cytotoxicity, antibody-dependent cellular phagocytosis and complement-dependent cytotoxicity effector functions in humans [11].

Tislelizumab is a humanised IgG4 monoclonal antibody with high affinity and specificity for PD-1 that was engineered to minimise binding to FcγR on macrophages [12, 13]. As a single agent and in combination with chemotherapy, tislelizumab has been shown to be generally well tolerated and has demonstrated anti-tumour activity in patients with solid tumours [14–17]. Tislelizumab is approved in China for first- to third-line treatment of non-small cell lung cancer and has conditional approval for second-line treatment of urothelial carcinoma (UC), second-line (or later) treatment of hepatocellular carcinoma and third-line treatment of classical Hodgkin’s lymphoma, with many more cancer indications currently under investigation [12, 18]. Several studies of tislelizumab have already shown promising evidence of anti-tumour activity with a manageable safety and tolerability profile in solid tumours [14–17]. Although, as yet, there are no data to suggest tislelizumab is superior to other PD-1 inhibitors, the availability of this antibody provides an opportunity to explore novel treatment combinations for advanced solid tumours.

Combining immune checkpoint inhibitors may help counter resistance pathways and increase the sensitivity to PD-1/PD-L1 treatment, offering the potential for improved anti-tumour responses and patient outcomes [19, 20]. Compared with monotherapy, a meta-analysis found that combined doublet immunotherapy was associated with significantly better overall survival (OS) and substantial improvements in progression-free survival (PFS) and disease control rate [21]. Beyond the US FDA-approved combinations of a PD-1 inhibitor with the cytotoxic T-lymphocyte antigen 4 (CTLA-4) inhibitor ipilimumab or the lymphocyte activation gene-3 inhibitor relatlimab [22, 23], a wide variety of other combinations are under investigation [19, 20]. Anti-PD-1 antibodies, such as tislelizumab, block the binding of PD-1 to both PD-L1 and PD-L2, thus inhibiting PD-1-mediated negative signalling in T cells [12]. However, PD-L1 also interacts with CD80 (B7-1), which can exert inhibitory effects on immunity [24, 25]. This interaction between PD-L1 and CD80 is blocked by PD-L1 inhibitors, which in turn release inhibitory signals to T cells [10]. The more complete inhibition of PD-1/PD-L1/PD-L2 pathways offered by the combination of PD-1 and PD-L1 inhibitors may elicit a stronger anti-tumour effect than inhibition of single components of the pathway alone, and warrants investigation. Clinical trials are currently exploring this PD-1/PD-L1 combination approach [26–28]. Here, we report results from a Phase 1/2 study of the novel PD-L1 inhibitor BGB-A333 alone and in combination with tislelizumab, in patients with advanced solid tumours (NCT03379259).

## Methods

### Study design

This was a Phase 1/2, open-label, multicentre, non-randomised study to investigate the safety, tolerability, pharmacokinetics (PK) and preliminary anti-tumour activity of BGB-A333 alone and in combination with tislelizumab in patients with advanced solid tumours.

**Fig. 1:**
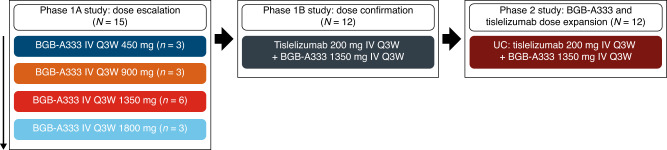
Study design. IV intravenous, Q3W every 3 weeks, UC urothelial carcinoma.

The study consisted of two phases, each comprising two parts (Fig. 1). Phase 1 of the study investigated the safety and tolerability of BGB-A333 alone and in combination with tislelizumab: in Phase 1a (dose escalation), patients received single-agent BGB-A333 intravenously (IV) every 3 weeks (Q3W) at increasing doses (450, 900, 1350 and 1800 mg), following a 3 + 3 design to establish the recommended Phase 2 dose (RP2D) of BGB-A333. The selected starting dose of 450 mg and range of additional dose levels (900, 1350 and 1800 mg) were based on safety data from monkeys and the projected human efficacious dose from preclinical studies in mice. Dose escalation, modification and selection decisions in Phase 1b and Phase 2 were determined after reviewing all available safety, efficacy, PK and exploratory data.

Using the dose of BGB-A333 determined in the dose-escalation phase (1350 mg), and the standard dose of tislelizumab that has been established previously [17], Phase 1b (combination dose confirmation) explored the safety and tolerability of IV BGB-A333 in combination with IV tislelizumab (200 mg Q3W).

Following the determination of the RP2D as 1350 mg, Phase 2 (combination dose expansion) evaluated the anti-tumour activity, safety and tolerability of BGB-A333 in combination with tislelizumab. Phase 2 (combination dose expansion) enroled patients with UC only, chosen based on data from Phase 1a.

### Patient population

Eligible patients were 18 years of age or older with at least one measurable lesion as defined per Response Evaluation Criteria in Solid Tumors (RECIST) v1.1 [29], an Eastern Cooperative Oncology Group performance status of 0–1 and adequate organ function. In Phase 1 only, patients with histologically or cytologically confirmed advanced or metastatic, unresectable solid tumours who had progressed during or after standard therapy or for which treatment was not available, not tolerated or refused were included. In Phase 2 (combination dose expansion) only, patients with locally advanced and metastatic UC who had progressed during or after treatment with platinum-based chemotherapy or who could not tolerate platinum-based chemotherapy were included.

Key exclusion criteria included prior therapy with an anti-PD-1 or anti-PD-L1 therapy; active leptomeningeal disease or uncontrolled brain metastasis; active autoimmune diseases or history of autoimmune diseases that may relapse; any other active malignancy in the 2 years before the first dose of study treatment (except for any locally recurring cancer treated curatively); any condition that required systemic treatment with either corticosteroids or other immuno-suppressive medication ≤14 days before administration of study treatment; significant pulmonary disease or history of interstitial lung disease, non-infectious pneumonitis or uncontrolled diseases including pulmonary fibrosis and acute lung diseases; and severe chronic or active infections requiring systemic therapy. Full eligibility criteria are described in the Supplementary Methods.

### Interventions

Per the initial dosing regimen, BGB-A333 and tislelizumab were administered IV on Day 1 of each 21-day cycle (Q3W). For study arms involving BGB-A333 monotherapy, the infusion of BGB-A333 was administered over 60 (±5) min. If the drug was well tolerated in the first three cycles, on Cycle 4 Day 1 and subsequent cycles, BGB-A333 could be administered over 30 (±5) min.

For study arms requiring combination treatment, in Cycles 1 and 2, tislelizumab was administered over 60 (±5) min followed by the administration of BGB-A333 over 60 (±5) min. If infusions were well tolerated in the first two cycles, on Cycle 3 Day 1, tislelizumab could be administered over 30 (±5) min followed by the administration of BGB-A333 over 60 (±5) min. If infusions of tislelizumab and BGB-A333 were well tolerated in the first three cycles, on Cycle 4 Day 1 and subsequent cycles, tislelizumab could be administered over 30 (±5) min followed by the administration of BGB-A333 over 30 (±5) min.

Dose reduction was not permitted for either BGB-A333 or tislelizumab; criteria for dose delay or modification are described in the Supplementary Methods. Patients received study drugs until they were no longer considered to be achieving clinical benefit, experienced unacceptable toxicity, or withdrew informed consent.

### Endpoints and assessments

The primary objectives for Phase 1a (dose escalation for BGB-A333 monotherapy) and Phase 1b (dose confirmation for BGB-A333 and tislelizumab combination) were to determine the RP2D for BGB-A333 alone and in combination with tislelizumab, and to assess the safety and tolerability of BGB-A333 alone and in combination with tislelizumab in patients with advanced solid tumours. The primary objective for Phase 2 (combination dose expansion) was to evaluate the investigator-assessed ORR per RECIST v1.1 [29] of BGB-A333 in combination with tislelizumab in patients with UC.

The secondary objectives for Phase 1a and Phase 1b were to assess the preliminary anti-tumour activity, PK and host immunogenicity (assessed via the incidence of antidrug antibodies [ADAs]) of BGB-A333 alone and in combination with tislelizumab. The secondary objectives for Phase 2 (combination dose expansion) were to assess other tumour assessment outcomes, specific duration of response (DoR), PFS and disease control rate per RECIST v1.1 [29]; to characterise the safety and tolerability of BGB-A333 in combination with tislelizumab; to characterise the PK of BGB-A333 in combination with tislelizumab; and to assess host immunogenicity to BGB-A333 and tislelizumab.

Exploratory endpoints for Phase 1b and 2 (combination dose expansion) included potential predictive biomarkers in archival and/or fresh tumour tissue and association with response to BGB-A333 alone and in combination with tislelizumab.

### Biomarker evaluation

Patients’ PD-L1 status was evaluated using the VENTANA PD-L1 (SP263) Assay (Ventana Medical Systems, Inc., Tuscon, Arizona, USA) in tumour samples collected at screening. Archival tissue samples were used if available, otherwise, a fresh biopsy was recommended. PD-L1-positive status was defined as ≥1% of tumour cells (TCs) with PD-L1 expression, except in the Phase 2 (combination dose expansion) UC cohort. In the Phase 2 UC cohort, patients were considered to be PD-L1-positive if immune cells (ICs) involved >1% of the tumour area and ≥25% of TCs or ICs had PD-L1 expression, or if ICs involved ≤1% of the tumour area and ≥25% of TCs or 100% of ICs expressed PD-L1, consistent with the approach used in a previously reported Phase 2 trial of tislelizumab monotherapy in locally advanced/metastatic UC [30].

### Statistical analyses

The intent-to-treat population included all patients who received at least one dose of the study drug and formed the population assessed in the safety and efficacy analyses. Dose-limiting toxicities (DLTs) were determined from the DLT-evaluable population for BGB-A333 monotherapy, which included patients who received at least two-thirds of the assigned dose of BGB-A333 during the DLT observation period (i.e. within 21 days of the first dose of BGB-A333) and had sufficient safety evaluation, or patients who experienced a DLT within the DLT observation period. DLTs were assessed among evaluable patients within 21 days after the first dose of BGB-A333. The PK analysis population included all patients with valid PK sampling after treatment with the study drug. Missing data were not imputed unless specified. SAS v.9.3 or higher was used for statistical analyses. Descriptive statistics were used to summarise all study data. Median time and 95% confidence interval (CI) for PFS were estimated by the Kaplan–Meier method.

### Reporting summary

Further information on research design is available in the Nature Portfolio Reporting Summary linked to this article.

## Results

### Baseline characteristics

Between November 2017 and September 2020, 12 study centres in Australia, New Zealand and Spain enroled 39 patients across Phase 1a (*N* = 15), Phase 1b (*N* = 12) and Phase 2 (*N* = 12) of the study, all of whom received at least one dose of study drug. Patient demographics and baseline characteristics are summarised in Table 1A and Table 1B. In Phase 1a, the median age of patients was 63.0 years (range: 30–70 years). The majority of patients were female and White or Caucasian. Five patients (33.3%) had a positive PD-L1 status. In Phase 1b and Phase 2 (combination dose expansion), the median age of patients was 71.0 years (range: 46–78 years). The majority of patients were male and White or Caucasian. In Phase 1b and Phase 2, four patients (33.3%) and six patients (50.0%), respectively, had a positive PD-L1 status. In all Phases, all patients had metastatic disease at study entry, most commonly involving the lymph nodes (66.7% in Phase 1a, and 54.2% in Phase 1b and Phase 2), lungs (33.3% in Phase 1a, and 41.7% in Phase 1b and Phase 2), peritoneum (33.3% in Phase 1a, and 12.5% in Phase 1b and Phase 2) and liver (26.7% in Phase 1a and 33.3%% in Phase 1b and Phase 2). Almost all patients had received ≥1 systemic therapy (Phase 1a: 93.3% of patients; Phase 1b and Phase 2: 87.5% of patients), and many had received ≥2 prior systemic therapies, particularly in Phase 1a (Phase 1a: 53.3% of patients; Phase 1b and Phase 2: 29.2% of patients).

**Table 1:** Patient demographics and baseline characteristics, intent-to-treat population, (A) Phase 1a; (B) Phase 1b and Phase 2.

(A)					
	BGB-A333 450 mg (*n* = 3)	BGB-A333 900 mg (*n* = 3)	BGB-A333 1350 mg (*n* = 6)	BGB-A333 1800 mg (*n* = 3)	Total (*N* = 15)
Median age, years (range)	58.0 (48–62)	63.0 (48–70)	65.5 (30–67)	66.0 (39–69)	63.0 (30–70)
Female, *n* (%)	2 (66.7)	2 (66.7)	4 (66.7)	2 (66.7)	10 (66.7)
Race, *n* (%)					
White or Caucasian	3 (100.0)	3 (100.0)	6 (100.0)	2 (66.7)	14 (93.3)
Asian	0 (0.0)	0 (0.0)	0 (0.0)	1 (33.3)	1 (6.7)
ECOG performance status, *n* (%)					
0	3 (100.0)	1 (33.3)	2 (33.3)	3 (100.0)	9 (60.0)
1	0 (0.0)	2 (66.7)	4 (66.7)	0 (0.0)	6 (40.0)
PD-L1 status, *n* (%)^a^					
Positive	0 (0.0)	2 (66.7)	2 (33.3)	1 (33.3)	5 (33.3)
Negative	2 (66.7)	1 (33.3)	3 (50.0)	2 (66.7)	8 (53.3)
Missing	1 (33.3)	0 (0.0)	1 (16.7)	0 (0.0)	2 (13.3)
Metastatic sites, *n* (%)					
Lymph nodes	3 (100.0)	1 (33.3)	5 (83.3)	1 (33.3)	10 (66.7)
Lung	1 (33.3)	1 (33.3)	2 (33.3)	1 (33.3)	5 (33.3)
Peritoneum	1 (33.3)	1 (33.3)	2 (33.3)	1 (33.3)	5 (33.3)
Liver	0 (0.0)	0 (0.0)	3 (50.0)	1 (33.3)	4 (26.7)
Bone	0 (0.0)	1 (33.3)	0 (0.0)	2 (66.7)	3 (20.0)
Soft tissue	2 (66.7)	0 (0.0)	0 (0.0)	0 (0.0)	2 (13.3)
Brain	1 (33.3)	0 (0.0)	0 (0.0)	0 (0.0)	1 (6.7)
Muscle	1 (33.3)	0 (0.0)	0 (0.0)	0 (0.0)	1 (6.7)
Other^b^	2 (66.7)	1 (33.3)	1 (16.7)	0 (0.0)	4 (26.7)
Prior lines of systemic treatment, *n* (%)^c^					
1	0 (0.0)	1 (33.3)	3 (50.0)	2 (66.7)	6 (40.0)
2	2 (66.7)	1 (33.3)	1 (16.7)	0 (0.0)	4 (26.7)
≥3	1 (33.3)	0 (0.0)	2 (33.3)	1 (33.3)	4 (26.7)
Latest type of prior systemic treatment, *n* (%)					
Adjuvant	0 (0.0)	0 (0.0)	1 (16.7)	0 (0.0)	1 (6.7)
Locally advanced	0 (0.0)	1 (33.3)	0 (0.0)	2 (66.7)	3 (20.0)
Metastatic	3 (100.0)	1 (33.3)	5 (83.3)	1 (33.3)	10 (66.7)

(B)			
	Phase 1b BGB-A333 1350 mg + tislelizumab 200 mg (*N* = 12)	Phase 2 UC cohort BGB-A333 1350 mg + tislelizumab 200 mg (*N* = 12)	Total (*N* = 24)
Median age, years (range)	72.0 (48–76)	69.5 (46–78)	71.0 (46–78)
Female, *n* (%)	7 (58.3)	1 (8.3)	8 (33.3)
Race, *n* (%)			
White or Caucasian	12 (100.0)	10 (83.3)	22 (91.7)
Native Hawaiian or other Pacific Islander	0 (0.0)	1 (8.3)	1 (4.2)
Other	0 (0.0)	1 (8.3)	1 (4.2)
ECOG performance status, *n* (%)			
0	6 (50.0)	6 (50.0)	12 (50.0)
1	6 (50.0)	6 (50.0)	12 (50.0)
PD-L1 status, *n* (%)^a^			
Positive	4 (33.3)	6 (50.0)	10 (41.7)
Negative	7 (58.3)	6 (50.0)	13 (54.2)
Missing	1 (8.3)	0 (0.0)	1 (4.2)
Metastatic sites, *n* (%)			
Lymph nodes	6 (50.0)	7 (58.3)	13 (54.2)
Lung	7 (58.3)	3 (25.0)	10 (41.7)
Liver	7 (58.3)	1 (8.3)	8 (33.3)
Soft tissue	4 (33.3)	0 (0.0)	4 (16.7)
Peritoneum	2 (16.7)	1 (8.3)	3 (12.5)
Bone	1 (8.3)	1 (8.3)	2 (8.3)
Muscle	0 (0.0)	1 (8.3)	1 (4.2)
Kidney	0 (0.0)	1 (8.3)	1 (4.2)
Prior lines of systemic treatment, *n* (%)^c^			
1	4 (33.3)	10 (83.3)	14 (58.3)
2	4 (33.3)	1 (8.3)	5 (20.8)
≥3	1 (8.3)	1 (8.3)	2 (8.3)
Latest type of prior systemic treatment, *n* (%)			
Adjuvant	3 (25.0)	4 (33.3)	7 (29.2)
Locally advanced	0 (0.0)	0 (0.0)	0 (0.0)
Metastatic	6 (50.0)	7 (58.3)	13 (54.2)

*ECOG* Eastern Cooperative Oncology Group, *PD-L1* programmed death-ligand 1, *UC* urothelial carcinoma.

^a^The PD-L1 assay was performed on tissue samples from the 39 subjects enroled. In Phase 1, 27 samples were archival samples and there were no fresh samples. In Phase 2, 11 samples were archival samples and 1 sample was a fresh.

^b^’Other’ includes ovaries (sigmoid deposit), pelvis, pleura, spleen and pancreas.

^c^Missing information for one patient in Phase 1a (BGB-A333 900 mg arm), and for three patients in the Phase 1b cohort.

### Safety

#### Phase 1a (BGB-A333 dose escalation)

In Phase 1a, 12 patients (80.0%) experienced a treatment-emergent adverse event (TEAE) (Table 2A). Five patients (33.3%) experienced at least one Grade 3 or 4 TEAE, with the highest incidence observed for gastrointestinal disorders (20.0%, including dysphagia, oral lichenoid reaction, small intestinal obstruction [each *n* = 1]) and infections and infestations (13.3%, including respiratory tract infection and vestibular neuronitis [each *n* = 1]). Similarly, five patients (33.3%) experienced at least one serious TEAE, with the highest incidence observed for gastrointestinal disorders (13.3%, including dysphagia and small intestinal obstruction [each *n* = 1]) and infections and infestations (13.3%, including respiratory tract infection and vestibular neuronitis [each *n* = 1]). Other serious TEAEs included tumour haemorrhage and pneumonia aspiration (each *n* = 1). No TEAEs led to death. One patient (6.7%) experienced a TEAE that led to permanent discontinuation of treatment (oral lichenoid reaction). Dose delays and/or interruptions due to TEAEs were reported in six patients (40.0%). The most commonly reported TEAEs included fatigue, nausea and vomiting (four patients [26.7%] each) and myalgia (three patients [20.0%]) (Table 3). Immune-mediated TEAEs were reported in three patients (20.0%); of these, two (13.3%) experienced an immune-mediated AE that was considered Grade 3 or higher (oral lichenoid reaction and rash maculo-papular [each *n* = 1]) (Table 2A). The other immune-mediated TEAE was pneumonitis (Grade 2, *n* = 1). None of the reported immune-mediated TEAEs was considered serious. No DLTs were reported in Phase 1a and the Safety Monitoring Committee had no safety concerns regarding BGB-A333 as a monotherapy at any of the dose levels evaluated in Phase 1a. Eight patients (53.3%) experienced at least one treatment-related AE (TRAE), with the most common being fatigue in three patients (20.0%), and nausea, back pain and myalgia each reported in two patients (13.3% each). Two patients (13.3%) experienced one TRAE classed as Grade 3 or higher, including oral lichenoid reaction and rash maculo-papular (each *n* = 1). No serious TRAEs were observed (Table 2A).

**Table 2:** Overview of treatment-emergent adverse events, intent-to-treat population, (A) Phase 1a; (B) Phase 1b and Phase 2.

(A)					
	BGB-A333 450 mg (*n* = 3)	BGB-A333 900 mg (*n* = 3)	BGB-A333 1350 mg (*n* = 6)	BGB-A333 1800 mg (*n* = 3)	Total (*N* = 15)
Patients with at least one TEAE, *n* (%)	1 (33.3)	2 (66.7)	6 (100.0)	3 (100.0)	12 (80.0)
Treatment-related TEAE	1 (33.3)	1 (33.3)	4 (66.7)	2 (66.7)	8 (53.3)
Grade 3 and 4 TEAE, *n* (%)^a^	0 (0.0)	1 (33.3)	3 (50.0)	1 (33.3)	5 (33.3)
Treatment-related ≥ Grade 3 TEAE	0 (0.0)	1 (33.3)	1 (16.7)	0 (0.0)	2 (13.3)
Serious TEAE, *n* (%)	0 (0.0)	1 (33.3)	3 (50.0)	1 (33.3)	5 (33.3)
Treatment-related serious TEAE	0 (0.0)	0 (0.0)	0 (0.0)	0 (0.0)	0 (0.0)
TEAE leading to death, *n* (%)	0 (0.0)	0 (0.0)	0 (0.0)	0 (0.0)	0 (0.0)
TEAE leading to permanent treatment discontinuation, *n* (%)	0 (0.0)	0 (0.0)	1 (16.7)	0 (0.0)	1 (6.7)
TEAE leading to dose modification, *n* (%)	0 (0.0)	1 (33.3)	3 (50.0)	2 (66.7)	6 (40.0)
TEAE leading to dose interruption	0 (0.0)	1 (33.3)	3 (50.0)	2 (66.7)	6 (40.0)
Immune-mediated AE, *n* (%)	0 (0.0)	1 (33.3)	2 (33.3)	0 (0.0)	3 (20.0)
Immune-mediated AE ≥ Grade 3	0 (0.0)	1 (33.3)	1 (16.7)	0 (0.0)	2 (13.3)
Infusion-related reaction, *n* (%)	1 (33.3)	0 (0.0)	0 (0.0)	1 (33.3)	2 (13.3)
Infusion-related reaction ≥ Grade 3	0 (0.0)	0 (0.0)	0 (0.0)	0 (0.0)	0 (0.0)
Dose-limiting toxicity event, *n* (%)	0 (0.0)	0 (0.0)	0 (0.0)	0 (0.0)	0 (0.0)

(B)			
	Phase 1b BGB-A333 1350 mg + tislelizumab 200 mg (*N* = 12)	Phase 2 UC cohort BGB-A333 1350 mg + tislelizumab 200 mg (*N* = 12)	Total (*N* = 24)
Patients with at least one TEAE, *n* (%)	12 (100.0)	12 (100.0)	24 (100.0)
Treatment-related TEAE	7 (58.3)	5 (41.7)	12 (50.0)
BGB-A333-related TEAE	6 (50.0)	5 (41.7)	11 (45.8)
Tislelizumab-related TEAE	7 (58.3)	5 (41.7)	12 (50.0)
Grade ≥ 3 TEAE, *n* (%)	7 (58.3)	4 (33.3)	11 (45.8)
Treatment-related ≥Grade 3 TEAE	3 (25.0)	2 (16.7)	5 (20.8)
BGB-A333-related ≥Grade 3 TEAE	3 (25.0)	2 (16.7)	5 (20.8)
Tislelizumab-related ≥Grade 3 TEAE	3 (25.0)	2 (16.7)	5 (20.8)
Serious TEAE, *n* (%)	5 (41.7)	3 (25.0)	8 (33.3)
Treatment-related serious TEAE	2 (16.7)	1 (8.3)	3 (12.5)
BGB-A333-related serious TEAE	2 (16.7)	1 (8.3)	3 (12.5)
Tislelizumab-related serious TEAE	2 (16.7)	1 (8.3)	3 (12.5)
TEAE leading to death, *n* (%)	1 (8.3)	1 (8.3)	2 (8.3)
BGB-A333-related TEAE leading to death	1 (8.3)	0 (0.0)	1 (4.2)
Tislelizumab-related TEAE leading to death	1 (8.3)	0 (0.0)	1 (4.2)
TEAE leading to permanent treatment discontinuation, *n* (%)	4 (33.3)	2 (16.7)	6 (25.0)
TEAE leading to dose modification, *n* (%)	1 (8.3)	2 (16.7)	3 (12.5)
TEAE leading to dose interruption	1 (8.3)	2 (16.7)	3 (12.5)
TEAE leading to dose reduction	0 (0.0)	0 (0.0)	0 (0.0)
Immune-mediated AE, *n* (%)	3 (25.0)	2 (16.7)	5 (20.8)
Immune-mediated AE ≥ Grade 3	2 (16.7)	1 (8.3)	3 (12.5)
Infusion-related reaction, *n* (%)	0 (0.0)	0 (0.0)	0 (0.0)
Infusion-related reaction ≥Grade 3	0 (0.0)	0 (0.0)	0 (0.0)
Dose-limiting toxicity event, *n* (%)	1 (8.3)	0 (0.0)	1 (4.2)

*AE* adverse event, *TEAE* treatment-emergent adverse event, *UC* urothelial carcinoma.

^a^No Grade 5 TEAEs occurred in Phase 1a.

**Table 3:** Treatment-emergent adverse events by preferred term reported in ≥10% of total patients, intent-to-treat population, (A) Phase 1a; (B) Phase 1b and Phase 2.

(A)					
Preferred term, *n* (%)	BGB-A333 450 mg (*n* = 3)	BGB-A333 900 mg (*n* = 3)	BGB-A333 1350 mg (*n* = 6)	BGB-A333 1800 mg (*n* = 3)	Total (*N* = 15)
Any TEAE	1 (33.3)	2 (66.7)	6 (100.0)	3 (100.0)	12 (80.0)
Fatigue	0 (0.00)	1 (33.3)	1 (16.7)	2 (66.7)	4 (26.7)
Nausea	1 (33.3)	0 (0.0)	3 (50.0)	0 (0.0)	4 (26.7)
Vomiting	0 (0.0)	1 (33.3)	3 (50.0)	0 (0.0)	4 (26.7)
Myalgia	0 (0.0)	1 (33.3)	0 (0.0)	2 (66.7)	3 (20.0)
Abdominal pain upper	0 (0.0)	0 (0.0)	1 (16.7)	1 (33.3)	2 (13.3)
Anaemia	0 (0.0)	1 (33.3)	0 (0.0)	1 (33.3)	2 (13.3)
Back pain	0 (0.0)	1 (33.3)	0 (0.0)	1 (33.3)	2 (13.3)
Cough	0 (0.0)	1 (33.3)	0 (0.0)	1 (33.3)	2 (13.3)
Diarrhoea	0 (0.0)	0 (0.0)	1 (16.7)	1 (33.3)	2 (13.3)
Headache	0 (0.0)	1 (33.3)	0 (0.0)	1 (33.3)	2 (13.3)
Hypercalcaemia	0 (0.0)	1 (33.3)	0 (0.0)	1 (33.3)	2 (13.3)
Lower respiratory tract infection	0 (0.0)	0 (0.0)	2 (33.3)	0 (0.0)	2 (13.3)
Oral candidiasis	0 (0.0)	0 (0.0)	1 (16.7)	1 (33.3)	2 (13.3)
Pelvic pain	1 (33.3)	0 (0.0)	1 (16.7)	0 (0.0)	2 (13.3)
Pyrexia	0 (0.0)	1 (33.3)	0 (0.0)	1 (33.3)	2 (13.3)
Rash	0 (0.0)	1 (33.3)	0 (0.0)	1 (33.3)	2 (13.3)
Rash, macro-papular	0 (0.0)	1 (33.3)	0 (0.0)	1 (33.3)	2 (13.3)

(B)			
Preferred term, *n* (%)	Phase 1b BGB-A333 1350 mg + tislelizumab 200 mg (*N* = 12)	Phase 2 UC cohort BGB-A333 1350 mg + tislelizumab 200 mg (*N* = 12)	Total (*N* = 24)
Any TEAE	12 (100.0)	12 (100.0)	24 (100.0)
Diarrhoea	4 (33.3)	2 (16.7)	6 (25.0)
Anaemia	3 (25.0)	1 (8.3)	4 (16.7)
Fatigue	2 (16.7)	2 (16.7)	4 (16.7)
Nausea	4 (33.3)	0 (0.0)	4 (16.7)
Pain in extremity	2 (16.7)	2 (16.7)	4 (16.7)
Asthenia	0 (0.00)	3 (25.0)	3 (12.5)
Coug	3 (25.0)	0 (0.00)	3 (12.5)
Insomnia	1 (8.3)	2 (16.7)	3 (12.5)
Musculoskeletal chest pain	2 (16.7)	1 (8.3)	3 (12.5)
Pruritis	2 (16.7)	1 (8.3)	3 (12.5)
Rash, maculo-papular	2 (16.7)	1 (8.3)	3 (12.5)

AEs were coded using MedDRA Version 23.0 and graded using CTCAE Version 4.03.

For each row category, a patient with two or more AEs in that category was counted only once.

*AE* adverse event, *CTCAE* Common Terminology Criteria for Adverse Events, *MedDRA* Medical Dictionary for Regulatory Activities, *TEAE* treatment-emergent adverse event, *UC* urothelial carcinoma.

#### Phase 1b and Phase 2 (combination dose expansion)

The RP2D for BGB-A333 of 1350 mg was selected for administration alongside tislelizumab in Phase 1b and Phase 2. In Phase 2 (combination dose expansion), only one cohort was opened for dose expansion, with a total of 12 patients treated in the metastatic UC arm.

In Phase 1b and Phase 2 (combination dose expansion), all 24 patients (100.0%) experienced a TEAE (Table 2B). Eleven patients (45.8%) experienced at least one Grade 3 or 4 TEAE, with the highest incidence observed for anaemia reported in 2 patients (8.3%). Other common Grade 3 or 4 TEAEs by system organ class were infections and infestations (12.5% [*n* = 3] including parainfluenza virus infection, pneumonia, skin bacterial infection and urinary tract infection, each *n* = 1), investigations (8.3% [*n* = 2] including aspartate aminotransferase increased and blood creatinine phosphokinase increased, each *n* = 1), and renal and urinary disorders (8.3% [*n* = 2] including acute kidney injury and haematuria, each *n* = 1). At least one serious TEAE was reported in eight patients (33.3%), with the highest incidence observed for renal and urinary disorders (8.3% [*n* = 2], including acute kidney injury and haematuria, each *n* = 1). Two patients (8.3%) experienced TEAEs that led to death. One death was due to acute kidney injury and was considered immune-mediated and related to both BGB-A333 and tislelizumab. The other death was due to generalised oedema and multiple organ dysfunction syndrome; this was considered unrelated to either study drug. Six patients (25.0%) experienced TEAEs that led to permanent discontinuation of treatment. TEAEs leading to dose delays and/or interruptions were reported in three patients (12.5%). The most commonly reported TEAEs included diarrhoea (six patients [25.0%]); anaemia, fatigue, nausea and pain in extremities (four patients [16.7%] each) (Table 3B).

Immune-mediated TEAEs were reported in five patients (20.8%) (Table 2B). Three of these patients (12.5%) experienced at least one immune-mediated TEAE that was Grade 3 or higher, including hypophysitis, immune-mediated hepatitis, acute kidney injury and rash maculo-papular (each *n* = 1). The immune-mediated hepatitis was considered serious (Grade 4) but resolved, and the acute kidney injury (Grade 5) led to death.

TRAEs were reported in 12 patients (50.0%) in total (Table 2B); the most common were rash maculo-papular (12.5% [*n* = 3]), and diarrhoea, nausea, asthenia, fatigue, myalgia and pruritus (each 8.3% [*n* = 2]). Eleven patients (45.8%) experienced TRAEs related to BGB-A333, and 12 patients (50.0%) experienced TRAEs related to tislelizumab. Grade 3 or 4 TRAEs related to BGB-A333 and tislelizumab were reported in five patients (20.8%), and included fatigue, immune-mediated hepatitis, acute kidney injury, rash, hypophysitis and increased serum creatinine phosphokinase. Serious TRAEs were reported in three patients (12.5%), all of which were considered related to both BGB-A333 and tislelizumab (Table 2B).

### Pharmacokinetics

Serum concentrations of BGB-A333 dropped exponentially after IV administration. PK parameters following BGB-A333 dosing in the first and fifth cycles are summarised in Table S1. The increase in BGB-A333 exposures, as measured by observed maximum concentration (C_max_) and area under the concentration-time curve from 0 to 21 days post-dose (AUC_0–21 day_), was approximately dose proportional from 450 to 1800 mg. Following BGB-A333 administration Q3W, PK exposures showed a ≤2-fold accumulation (1.8-fold or 1.3-fold accumulation for AUC_0–21 day_ or C_max_, respectively). The geometric mean (n, geometric coefficient of variation %) values of AUC_0–21 day_ at Cycles 1 and 5 were 3791 (*n* = 5, 14.5%) and 6352 (*n* = 4, 14.1%) μg•day/mL, respectively, at the BGB-A333 1350 mg dose level in Phase 1a. Steady-state exposures with BGB-A333 1350 mg Q3W as a monotherapy were similar to those with BGB-A333 1350 mg Q3W in combination with tislelizumab 200 mg Q3W (Fig. S1).

### Immunogenicity

Thirty-eight patients were considered evaluable for ADAs to BGB-A333 (treatment-emergent ADAs) as they had a baseline and ≥1 post-baseline ADA result. The incidence of treatment-emergent ADAs across all phases was 18.4% (7/38), with three patients (7.9%) testing positive for neutralising antibodies. No patients showed evidence of treatment-boosted ADAs. Of the seven patients with treatment-induced ADAs, four had persistent ADA responses (4/38; 10.5% of evaluable patients). There was no apparent effect of immunogenicity on the BGB-A333 PK profile (data not shown).

The incidence of ADAs to tislelizumab (treatment-emergent ADAs) in Phase 1b and Phase 2 (combination dose expansion) was 21.7% (5/23 evaluable patients who had baseline and ≥1 post-baseline ADA results), with two patients (8.7%) testing positive for neutralising antibodies. No patients showed evidence of treatment-boosted ADAs. Of the five patients with treatment-induced ADAs, two had persistent ADA responses (2/23; 8.7% of evaluable patients).

### Anti-tumour activity

#### Preliminary efficacy

In Phase 1a, five patients achieved an objective response (complete response [CR] or partial response [PR]) (Table S2A), which included two patients with squamous cell carcinoma, one patient with cervical cancer, one patient with UC and one patient with breast cancer. Among the patients with responses, two were observed in patients with lymph node-only disease. The median DoR was not reached.

**Fig. 2:**
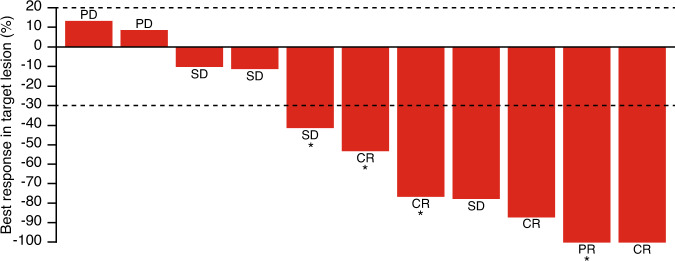
Best response: target lesion changes over time, Phase 2, safety analysis set. *Patients with lymph node-only disease. CR complete response, PD progressive disease, PR partial response, SD stable disease.

In Phase 1b, the ORR was 16.7% (*n* = 2/12; 95% CI: 2.09%, 48.41%) (two patients achieved a PR, one with squamous cell carcinoma and the other with colorectal cancer) (Table S2B). The median DoR was not reached (Table 4). In Phase 2 (combination dose expansion), the ORR was 41.7% (*n* = 5/12; 95% CI: 15.17%, 72.33%) (Table S2B). In total, four patients (33.3%) achieved a CR (one patient with UC, two with bladder cancer and one with upper urothelial tract cancer) and one patient (8.3%) achieved a PR (bladder cancer). The median DoR was 9.6 months (95% CI: 6.0, not estimable [NE]) (Table 4). The best percentage change from baseline in the sum of target lesion diameters per investigator assessment for Phase 2 (combination dose expansion) is provided in Fig. 2. The majority of responses in Phase 1b and Phase 2 were seen in patients with lymph node-only disease (*n* = 4).

**Table 4:** Duration of response, Phase 1b and Phase 2, intent-to-treat population.

	Phase 1b BGB-A333 1350 mg + tislelizumab 200 mg (*N* = 12)	Phase 2 UC cohort BGB-A333 1350 mg + tislelizumab 200 mg (*N* = 12)
Number of responders	2	5
Number of patients with events, *n* (%)	0 (0.0)	3 (60.0)
PD	0 (0.0)	3 (60.0)
Death	0 (0.0)	0 (0.0)
Number of patients censored, *n* (%)	2 (100.0)	2 (40.0)
DoR, months		
Median (95% CI)	NE	9.6 (6.0, NE)
Q1 (95% CI)	NE	9.1 (6.0, 9.6)
Q3 (95% CI)	NE	NE (6.0, NE)
Event-free rate (95% CI)		
3 Month	100.0 (NE, NE)	100.0 (NE, NE)
6 Month	100.0 (NE, NE)	100.0 (NE, NE)
9 Month	100.0 (NE, NE)	80.0 (20.4, 96.9)
12 Month	NE	NE

DoR defined as time from first determination of an objective response per RECIST v1.1 [29] until the first documentation of disease progression or death, whichever occurred first.

*CI* confidence interval, *DoR* duration of response, *NE* not estimable, *PD* progressive disease, *RECIST* Response Evaluation Criteria in Solid Tumors, *UC* urothelial carcinoma.

#### Estimated progression-free survival

In Phase 1b, the median PFS was 4.7 months (95% CI: 1.5, NE) (Fig. S2A). The PFS event-free rates at 6 and 12 months were 46.3% and 27.8%, respectively. In Phase 2 (combination dose expansion), the median PFS was 6.1 months (95% CI: 1.9, 11.0) (Fig. S2A). The PFS event-free rates at 6 and 12 months were 66.7% and 11.1%, respectively.

#### Follow-up

In Phase 1a, all patients discontinued BGB-A333 treatment, the primary reason being disease progression (Fig. S3A). The median follow-up time on the study was 8.3 months (range: 1.5–27.5 months). In Phase 1b and Phase 2 (combination dose expansion), all 24 patients discontinued from BGB-A333 and tislelizumab treatment, the primary reason being disease progression (Fig. S3B). The median follow-up time on study was 8.8 months (range: 1.2–22.1 months).

### Biomarker evaluation

In Phase 1a, the ORR was numerically higher in PD-L1-positive patients than in PD-L1-negative patients (*n* = 4, 80% vs *n* = 1, 12.5%; Table S2), irrespective of the dose received. In Phase 1b, a similar trend of a numerically higher ORR (25.0% vs 14.3%) was observed in PD-L1-positive patients compared with PD-L1-negative patients. In the Phase 2 (combination dose expansion) cohort, the ORR in the PD-L1-positive group was 66.7% compared with 16.7% in the PD-L1-negative group (Table S2).

## Discussion

This study was an open-label, multicentre, non-randomised study to investigate the safety, tolerability, PK and preliminary anti-tumour activity of the anti-PD-L1 monoclonal antibody BGB-A333 alone and in combination with the anti-PD-1 monoclonal antibody tislelizumab in patients with advanced solid tumours. Treatment with BGB-A333 1350 mg Q3W, which was considered the RP2D, as monotherapy or in combination with tislelizumab 200 mg Q3W, was generally well tolerated with a manageable safety profile.

The safety profile of BGB-A333 in the Phase 1a study was consistent with the established profile of anti-PD-1/L1 therapies [31]. Such therapies are known to increase the risk of immune-mediated AEs [31], and combining two immunotherapies could compound the risk of immune-mediated AEs. However, reassuringly the incidence of both all-grade and ≥Grade 3 immune-mediated AEs was similar for BGB-A333 monotherapy in phase 1a (20.0% and 13.3%, respectively) and BGB-A333 plus tislelizumab combination therapy in Phase 1b/2 (20.8% and 12.5%, respectively). More broadly, the incidence of all-grade and ≥Grade 3 TRAEs with the combination (50.0% and 20.8%, respectively) was lower than that reported in a recent systematic review and meta-analysis of immunotherapy combination therapy studies (86.8% and 35.9%, respectively) [32]. Consistent with results in the present study for BGB-A333 plus tislelizumab, the meta-analysis found the most common all-grade TRAE in patients treated with immunotherapy combinations was fatigue [32]. Recently, the results of a Phase 2 trial evaluating the anti-PD-1 antibody MEDI0680 combined with anti-PD-L1 treatment durvalumab in patients with advanced or metastatic clear-cell renal cell carcinoma have been reported [27]. Accepting the limitation of cross-trial comparisons, this study reported a higher incidence of all-grade TRAEs with MEDI0680 plus durvalumab (92.9% of patients) than seen with BGB-A333 plus tislelizumab in the present study (50.0%), while the incidences of TEAEs leading to discontinuation of treatment were similar (23.8% and 25.0% of patients, respectively) [27].

The present study also provided preliminary evidence of the anti-tumour activity of BGB-A333 in combination with tislelizumab in PD-1/PD-L1 inhibitor naïve patients with advanced solid tumours, most of whom had received at least one prior line of systemic therapy. In the combination dose-expansion phase (Phase 2) in patients with UC, the confirmed ORR was 41.7% (5/12 patients), with four patients achieving CR and one patient achieving PR, and responses were durable. These preliminary data on the anti-tumour activity of this combination in patients with advanced UC are more encouraging than those reported with MEDI0680 plus durvalumab in patients with previously treated, immunotherapy-naïve, advanced renal cell carcinoma, which resulted in an ORR of only 16.7% [27]. The ORR for BGB-A333 plus tislelizumab combination therapy in the present study also compares favourably with findings of a Phase 2 study in patients with PD-L1-positive locally advanced or metastatic UC with progression during/following platinum-containing chemotherapy, in which tislelizumab monotherapy resulted in an ORR of 24% [30]. While this cross-trial comparison should be interpreted cautiously, the stronger anti-tumour response observed with the combination may be due to the overlapping mechanisms of BGB-A333 and tislelizumab increasing the inhibition of the PD-1/PD-L1/PD-L2 pathway and blocking more immuno-suppressive signals than tislelizumab alone.

All patients in the combination dose-expansion phase had UC. Our preliminary results on the anti-tumour activity of BGB-A333 plus tislelizumab combination therapy are encouraging in light of the continued unmet need for patients with advanced UC ineligible for first-line platinum-based chemotherapy, and for those requiring later lines of therapy [33]. Several anti-PD-1/PD-L1 monotherapies are currently US FDA-approved for the treatment of advanced UC in patients ineligible for platinum-containing chemotherapy [34, 35], or who have disease progression following platinum-containing chemotherapy [34, 36, 37]. However, a recent network meta-analysis of Phase 3 randomised controlled trials in metastatic UC found no survival benefit with anti-PD-1/PD-L1-based regimens versus chemotherapy as first-line therapy, either as monotherapy or in combination with chemotherapy [33]. In the second-line setting, of the anti-PD-1/PD-L1 monotherapy regimens studied (pembrolizumab or atezolizumab), only pembrolizumab demonstrated a survival benefit compared with chemotherapy, and neither significantly improved ORR [33]. Indeed, despite promising Phase 2 data for atezolizumab leading to accelerated approval as second-line therapy for advanced UC, atezolizumab did not improve OS versus chemotherapy in the subsequent Phase 3 IMvigor211 trial [38], leading to the voluntary withdrawal of the second-line indication [39, 40]. Similarly, in the Phase 3 DANUBE study, durvalumab failed to improve OS versus chemotherapy [41], again leading to the voluntary withdrawal of a previously granted indication for second-line treatment of advanced UC [39, 40]. Furthermore, it has been announced that the combination of first-line nivolumab plus ipilimumab failed to improve OS versus standard-of-care chemotherapy as first-line treatment of advanced UC in the Phase 3 CheckMate-901 trial [42]. In this context, results of the ongoing Phase 3 NILE study of durvalumab plus chemotherapy, with or without the anti-CTLA4 antibody tremelimumab, as first-line treatment for patients with advanced UC, are awaited with interest [43]. Given the mixed findings reported with immunotherapy in UC to date, there is clearly a need to identify regimens that offer greater efficacy.

In the present study, most responses in the combined dose-expansion Phase 2 part were seen in UC patients with lymph node-only disease. Metastasis to lymph nodes is a key step in the development of tumour cell immune tolerance [44]. As with other solid tumours [44], lymph node involvement in UC is associated with a poorer prognosis than cases without the nodal disease [45]. According to the latest US Surveillance, Epidemiology and End Results Program (SEER) data, the presence of nodal involvement in bladder cancer is associated with a pronounced decrease in 5-year disease-free survival, from 96.0% and 69.6% in patients with in situ or localised tumours, respectively, to 39.0% in those with spread to regional lymph nodes, and just 7.7% in those with metastasis to other sites [45]. In this context, the preliminary anti-tumour results for BGB-A333 plus tislelizumab combination therapy in this subgroup of patients are encouraging and support the continued investigation of this combination.

The exposures of BGB-A333 (AUC_0-21 day_ and C_max_) increased approximately dose-proportionally over the dose range tested in this study. Co-administration with tislelizumab did not alter BGB-A333 PK and vice versa; BGB-A333 did not affect the known steady-state exposure of tislelizumab. ADAs to BGB-A333 occurred across all phases, but there was no apparent effect of immunogenicity on the BGB-A333 PK profile.

### Study limitations

As these data are from a small non-randomised, open-label study, they have some inherent limitations. Response and PFS were assessed in a small, heterogeneous group of patients with a range of advanced solid tumours with different levels of PD-L1 expression. The UC group, although small, did demonstrate activity; however, it could not be definitively concluded that this was superior to what could be expected for single-agent PD-1 or PD-L1 treatment, given the small sample size.

## Conclusion

In this Phase 1/2 study, BGB-A333 alone or in combination with tislelizumab was generally well tolerated in patients with advanced solid tumours. The RP2D for BGB-A333 was estimated as 1350 mg Q3W. AEs reported with BGB-A333 were mostly mild to moderate in severity and consistent with those associated with other PD-L1 inhibitors. Preliminary anti-tumour activity was observed with BGB-A333 as both a single agent and in combination with tislelizumab. Co-administration of BGB-A333 with tislelizumab did not have a significant impact on the PK profile of either compound. Our findings in a small number of patients suggest that PD-1/PD-L1 combination therapy has the potential to augment the efficacy of PD-1 treatment with tislelizumab in selected tumour types; however, further investigation in larger-scale studies is warranted.

## Supplementary information

SUPPLEMENTARY INFORMATION

Reporting Summary Checklist

## Data Availability

The datasets used and/or analysed during the current study are available from the corresponding author on reasonable request.
