# Supplementary material for: A Phase 1/2 study of the PD-L1 inhibitor, BGB-A333, alone and in combination with the PD-1 inhibitor, tislelizumab, in patients with advanced solid tumours
Source: Br J Cancer. 2023 Feb 16;128(8):1418–28. doi: 10.1038/s41416-022-02128-3 (PMC10070264; doi:10.1038/s41416-022-02128-3)

**SUPPLEMENTARY INFORMATION

Methods**

**Full inclusion criteria**

Each patient eligible to participate in the study had to meet all of the following criteria:

1. Able to provide written informed consent and can understand and comply with the requirements of the study
2. Age ≥ 18 years on the day of signing the informed consent form (or the legal age of consent in the jurisdiction in which the study is taking place)
3. For Phase 1 only: histologically or cytologically confirmed advanced or metastatic, unresectable solid tumours with progression during or after standard therapy or for which treatment is not available, not tolerated or refused
4. For Phase 2 only: locally advanced and metastatic urothelial carcinoma with progression during or after treatment with platinum-based chemotherapy or intolerance of platinum-based chemotherapy

5. At least one measurable lesion as defined per Response Evaluation Criteria in Solid Tumors version 1.1 (RECIST v1.1)^25^

Note: The target lesion(s) selected must not have been previously treated with local therapy or the target lesion(s) selected that were within the field of prior local therapy had subsequently progressed as defined by RECIST v1.1^25^

6. Eastern Cooperative Oncology Group (ECOG) Performance Status ≤ 1

7. Adequate organ function as indicated by the following laboratory values:

a. Absolute neutrophil count (ANC) ≥ 1.5 × 109/L, platelets ≥ 75 × 109/L, haemoglobin ≥ 90 g/L. Note: Patients must not have required a blood transfusion or growth factor support ≤ 14 days before sample collection

b. Serum creatinine ≤ 1.5 × upper limit of normal (ULN), or estimated GFR ≥ 60 mL/min/1.73 m2 by Chronic Kidney Disease Epidemiology Collaboration equation

c. Aspartate transaminase (AST) and alanine aminotransferase (ALT) ≤ 3 × ULN

d. Serum total bilirubin ≤ 1.5 X ULN (total bilirubin must have been < 3 X ULN for patients with Gilberts syndrome)

e. For hepatocellular carcinoma (HCC) patients only, patient must have met the Child-Pugh A classification for liver function as assessed within 7 days before the first dose of study drug(s)

8. Females of childbearing potential must have been willing to use a highly effective method of birth control for the duration of the study, and ≥ 120 days after the last dose of study drug(s), and had a negative urine or serum pregnancy test ≤ 7 days of the first dose of study drug(s)

9. Non-sterile males must have been willing to use a highly effective method of birth control for the duration of the study and for ≥ 120 days after the last dose of study drug(s)

**Full exclusion criteria**

Patients who met any of the following criteria were not eligible to enrol:

1. Active leptomeningeal disease or uncontrolled brain metastasis. Patients with equivocal findings or with confirmed brain metastases were eligible for enrolment provided they were asymptomatic and radiologically stable without the need for corticosteroid treatment for at least 4 weeks prior to the first dose of study drug(s)
2. Active autoimmune diseases or history of autoimmune diseases that may relapse

Note: Patients with the following diseases were not excluded and could proceed to further screening:

a. Controlled type 1 diabetes

b. Hypothyroidism (provided it was managed with hormone replacement therapy only)

c. Controlled celiac disease

d. Skin diseases not requiring systemic treatment (eg, vitiligo, psoriasis, alopecia)

e. Any other disease that is not expected to recur in the absence of external triggering factors

1. Any active malignancy ≤ 2 years before the first dose of study drug(s), except for the specific cancer that was under investigation in this study and any locally recurring cancer that had been treated curatively (eg, resected basal or squamous cell skin cancer, superficial bladder cancer, carcinoma in situ of the cervix or breast)
2. Any condition that required systemic treatment with either corticosteroids (> 10 mg daily of prednisone or equivalent) or other immunosuppressive medication ≤ 14 days before administration of study drug

Note: Patients who were or had previously been on any of the following steroid regimens were not excluded:

a. Adrenal replacement steroid (dose ≤ 10 mg daily of prednisone or equivalent)

b. Topical, ocular, intra-articular, intranasal, or inhalational corticosteroid with minimal systemic absorption

c. Short course (≤ 7 days) of corticosteroid prescribed prophylactically (eg, for contrast dye allergy) or for the treatment of a non-autoimmune condition (eg, delayed-type hypersensitivity reaction caused by contact allergen)

1. Uncontrolled diabetes or Grade > 1 laboratory test abnormalities in potassium, sodium, or corrected calcium despite standard medical management
2. Significant pulmonary disease (ie, chronic obstructive pulmonary disease [COPD], emphysema or chronic bronchitis) or history of interstitial lung disease, non-infectious pneumonitis or uncontrolled diseases including pulmonary fibrosis, acute lung diseases, etc.
3. Severe chronic or active infections requiring systemic antibacterial, antifungal or antiviral therapy, including tuberculosis infection, etc. Note: antiviral therapy was permitted for patients with HCC
4. A known history of human immunodeficiency virus (HIV) infection
5. A known history of hepatitis B virus (HBV) or hepatitis C virus (HCV) infection, except for patients with HCC. Note: For patients with HCC only: patients with detectable hepatitis B surface antigen (HBsAg) or detectable HCV antibody at screening were excluded unless their HBV DNA titres < 500 IU/mL or HCV RNA polymerase chain reaction test, respectively, was negative. In addition, patients with detectable HBsAg or detectable HBV DNA were managed per treatment guidelines. Patients receiving antivirals at screening must have been treated for > 2 weeks prior to the first dose of study drug(s) and continued treatment for 6 months after the last dose of study drug(s)
6. Any major surgical procedure ≤ 28 days before the first dose of study drug(s)
7. Prior allogeneic stem cell transplantation or organ transplantation
8. Any of the following cardiovascular criteria:

a. Cardiac chest pain, defined as moderate pain that limits instrumental activities of daily living, ≤ 28 days before the first dose of study drug(s)

b. Symptomatic pulmonary embolism ≤ 28 days before the first dose of study drug(s)

c. Any history of acute myocardial infarction ≤ 6 months before the first dose of study drug(s)

d. Any history of heart failure meeting New York Heart Association Classification (NYHA) III or IV (Appendix 6) ≤ 6 months before the first dose of study drug(s)

e. Any event of ventricular arrhythmia Grade ≥ 2 in severity ≤ 6 months before the first dose of study drug(s)

f. Any history of cerebrovascular accident ≤ 6 months before the first dose of study drug(s)

g. Uncontrolled hypertension: systolic pressure ≥ 160 mmHg or diastolic pressure ≥ 100 mmHg despite anti-hypertension medications ≤ 28 days before the first dose of study drug(s)

h. Any episode of syncope or seizure ≤ 28 days before the first dose of study drug(s)

1. A history of severe hypersensitivity reactions to other monoclonal antibodies (mAbs)
2. Received any chemotherapy, radiotherapy, immunotherapy (eg, interleukin, interferon, thymoxin, etc.) or any investigational therapies within 28 days or 5 half-lives (whichever is shorter) of the first study drug(s) administration. Received any Chinese herbal medicine or Chinese patent medicines used to control cancer within 14 days of the first study drug administration
3. Toxicities (as a result of prior anticancer therapy) which have not recovered to baseline or stabilized, except for adverse events (AEs) not considered a likely safety risk (eg, alopecia, neuropathy, and specific laboratory abnormalities)
4. Was administered a live vaccine ≤ 4 weeks prior to study drug administration

Note: seasonal vaccines for influenza are generally inactivated vaccines and were allowed. Intranasal vaccines are live vaccines, and are not allowed

1. Underlying medical conditions, laboratory abnormality, or alcohol or drug abuse or dependence that, in the investigator’s opinion, was unfavourable for the administration of study drug or affected the explanation of drug toxicity AEs; or insufficient compliance during the study according to investigator’s judgement
2. Concurrent participation in another therapeutic clinical trial
3. Received prior therapy with an anti-programmed cell death protein-1 (PD-1), anti-programmed death ligand-1 (PD-L1), anti-PD-L2, or anti-cytotoxic T lymphocyte antigen-4 (CTLA-4) antibody (or any other antibody targeting T-cell co-stimulation pathways).

**Criteria for dose delay / modification**

*Criteria for dose modification:*

There was no dose reduction for BGB-A333 or tislelizumab within a given patient in this study.

In Phase 1A (BGB-A333 monotherapy), if patients progressed on BGB-A333 without other safety concerns, these patients could have been treated with a higher dose of BGB-A333 that was deemed to be well tolerated by the safety monitoring committee (SMC). The decision to modify the dose of BGB-A333 was discussed with the sponsor’s medical monitor and documented in the study records.

In Phase 1A, if patients progressed on BGB-A333 monotherapy without other safety concerns, these patients could have received combination of BGB-A333 and tislelizumab at doses that were deemed to be well tolerated by SMC. The decision to add tislelizumab to the regimen for patients treated with BGB-A333 alone was discussed with the sponsor’s medical monitor and documented in the study records.

*Criteria for dose delay:*

Dose delays or interruptions of less than 12 weeks were permitted. The tumour assessment schedule was not altered even if the administration of study drug(s) was delayed.

Every effort was made to administer the study drug(s) according to the planned dose and schedule. In the event of significant toxicities, dosing could have been delayed and/or reduced based on the guidelines provided below. Reasons for dose modifications or delays, the supportive measures taken, and the outcome were documented in the patient's chart and recorded in the electronic case report form (eCRF).

Patients could have temporarily suspended study treatment if they experienced toxicity that was considered related to study drug(s) and required a dose to be withheld. The patients must have resumed study drug treatment as soon as possible after the AEs recovered to baseline or Grade 1 (whichever was more severe) within 12 weeks after the last dose of study drug(s).

If the patient was unable to resume study drug treatment within 12 weeks after the last dose of study drug, then the patient was discontinued from study drug treatment.

In case a patient was benefiting from the study treatment while meeting the discontinuation criteria, resumption of study treatment could have occurred upon discussion and agreement with sponsor medical monitor.

**TABLES**

**Table S1.** Summary of PK parameters (geometric means) of BGB-A333 following single IV dose (Cycle 1) and multiple IV doses (Cycle 5) of BGB-A333 in patients with advanced tumours (PK analysis set).

|  | **Phase 1a** | | | | **Phase 1b** |
| --- | --- | --- | --- | --- | --- |
|  | **BGB-A333**  **450 mg Q3W** | **BGB-A333**  **900 mg Q3W** | **BGB-A333**  **1350 mg Q3W** | **BGB-A333**  **1800 mg Q3W** | **BGB-A333**  **1350 mg Q3W** |
| **Cycle 1 Parameters**  **C_max_**, μg/mL | ***n* = 3**  163 (28.2) | ***n* = 3**  325 (53.4) | ***n* = 6**  457 (23.0) | ***n* = 3**  580 (26.9) | ***n* = 12**  439 (28.9) |
| **T_max_**, day | 0.07 (112) | 0.06 (10.6) | 0.06 (66.5) | 0.09 (82.9) | 0.12 (83.2) |
| **C_trough_**, μg/mL | 20.8 (26.2) | 40.5 (82.2) | 88.2 (26.0) | 79.2 (29.0) | 76.9 (31.0) |
| **t_last_**, day | 21.7 (2.69) | 18.4 (23.3) | 19.8 (17.2) | 22.0 (7.72) | 19.7 (33.3) |
| **AUC_0-21 day_**, μg•day/mL | 1089 (12.6) | 2904 (11.0)^a^ | 3791 (14.5)^b^ | 4109 (15.2) | 3455 (24.9)^c^ |
| **Cycle 5 Parameters**  **C_max_**, μg/mL | ***n* = 1**^d^  264 | ***n* = 2**  307 (50.4) | ***n* = 4**  632 (8.81) | ***n* = 1**^d^  682 | ***n* = 6**  608 (20.4) |
| **T_max_**, day | 0.02 | 0.02 (0.00) | 0.02 (0.00) | 0.02 | 0.02 (0.00) |
| **C_trough_**, μg/mL | 62.8 | 64.2 (28.4) | 167 (27.8) | 97.0 | 140 (45.3) |
| **T_last_**, day | 21.0 | 14.0 (0.00) | 21.0 (0.00) | 21.0 | 21.0 (0.00) |
| **AUC_0-21 day_**, µg·day/mL^e^ | 2421 | - | 6352 (14.1) | 5239 | 6357 (26.9) |

Geometric mean (geometric CV%) of PK parameters are reported. PK parameters were determined after BGB-A333 monotherapy in Phase 1a and after BGB-A333 and 200 mg Q3W tislelizumab combination therapy in Phase 1b.

^a^*n* = 2.

^b^*n* = 5.

^c^*n* = 11.

^d^Coefficient of variation percent not estimable when *n* = 1.

^e^If the PK sample was not collected at the 21-day timepoint after the first dose, AUC_0–21 day_ was not determined.

*AUC_0–21 day_* area under the concentration-time curve from 0 to 21 days post dose, *C_max_* observed maximum concentration, *C_trough_* last observed concentration prior to next dose, *CV%* coefficient of variation percent, *IV* intravenous, *PK* pharmacokinetic, *Q3W* every 3 weeks, *T_last_* time to last observed concentration, *T_max_* time to observed maximum concentration.

**Table S2.** Objective response rate and disease response by PD-L1 expression status, intent-to-treat population, (**A**) Phase 1a; (**B**) Phase 1b and Phase 2.

**A**

|  | **BGB-A333**  **450 mg**  **(*n* = 3)** | **BGB-A333**  **900 mg**  **(*n* = 3)** | **BGB-A333**  **1350 mg**  **(*n* = 6)** | **BGB-A333**  **1800 mg**  **(*n* = 3)** | **Total**  **(*N* = 15)** |
| --- | --- | --- | --- | --- | --- |
| **ORR^a^, n (%)** | 0 (0.0) | 1 (33.3) | 3 (50.0) | 1 (33.3) | 5 (33.3) |
| 95% CI | (0.00, 70.76) | (0.84, 90.57) | (11.81, 88.19) | (0.84, 90.57) | (11.82, 61.62) |
| **PD-L1 positive^b^, m** | 0 | 2 | 2 | 1 | 5 |
| n (%) | N/A | 1 (50.0) | 2 (100.0) | 1 (100.0) | 4 (80.0) |
| 95% CI | N/A | (1.26, 98.74) | (15.81, 100.00) | (2.50, 100.00) | (28.36, 99.49) |
| **PD-L1 negative, m** | 2 | 1 | 3 | 2 | 8 |
| n (%) | 0 (0.0) | 0 (0.0) | 1 (33.3) | 0 (0.0) | 1 (12.5) |
| 95% CI | (0.00, 84.19) | (0.00, 97.50) | (0.84, 90.57) | (0.00, 84.19) | (0.32, 52.65) |
| **PD-L1 missing, m** | 1 | 0 | 1 | 0 | 2 |
| n (%) | 0 (0.0) | N/A | 0 (0.0) | N/A | 0 (0.0) |
| 95% CI | (0.00, 97.50) | N/A | (0.00, 97.50) | N/A | (0.00, 84.19) |

^a^Objective response based on investigator tumour assessment of confirmed complete response or partial response according to RECIST v1.1.^25^

^b^Criteria for PD-L1 positive in Phase 1a: tumour cell ≥1%.

**B**

|  | **Overall response category** | **Phase 1b**  **BGB-A333 1350 mg +  tislelizumab 200 mg**  **(*N* = 12)** | **Phase 2 UC cohort**  **BGB-A333 1350 mg + tislelizumab 200 mg**  **(*N* = 12)** |
| --- | --- | --- | --- |
|  | **ORR^a^, n (%)** | 2 (16.7) | 5 (41.7) |
|  | 95% CI | (2.09, 48.41) | (15.17, 72.33) |
|  | **PD-L1 positive^b^, m** | 4 | 6 |
|  | n (%) | 1 (25.0) | 4 (66.7) |
|  | 95% CI | (0.63, 80.59) | (22.28, 95.67) |
|  | **PD-L1 negative, m** | 7 | 6 |
|  | n (%) | 1 (14.3) | 1 (16.7) |
|  | 95% CI | (0.36, 57.87) | (0.42, 64.12) |
|  | **PD-L1 missing, m** | 1 | 0 |
|  | n (%) | 0 (0.0) | N/A |
|  | 95% CI | (0.0, 97.50) | N/A |
|  | **BOR, n (%)** |  |  |
|  | CR | 0 (0.0) | 4 (33.3) |
|  | PR | 2 (16.7) | 1 (8.3) |
|  | SD | 5 (41.7) | 4 (33.3) |
|  | PD | 3 (25.0) | 2 (16.7) |
|  | NE | 2 (16.7) | 1 (8.3) |
|  | **DCR, n (%)** | 7 (58.3) | 9 (75.0) |
|  | 95% CI | (27.67, 84.83) | (42.81, 94.51) |
|  | **CBR^c^, n (%)** | 5 (41.7) | 8 (66.7) |
|  | 95% CI | (15.17, 72.33) | (34.89, 90.08) |

^a^Objective response based on investigator tumour assessment of confirmed complete response or partial response according to RECIST v1.1.^25^

^b^Criteria for PD-L1 positive in Phase 1b: tumour cell ≥1%; criteria for PD-L1 positive in Phase 2: tumour cell or immune cell ≥25% (if immune cells >1% of tumour area); or tumour cell ≥25% or immune cell 100% (if immune cells <1% of tumour area).

^c^CBR defined as the proportion of patients who achieved a BOR of CR, PR, and durable stable disease (BOR of stable disease with a duration of ≥24 weeks).

*BOR* best overall response, *CBR* clinical benefit rate, *CI* confidence interval, *CR* complete response, *DCR* disease control rate, *m* number of patients with PD-L1 positive/negative/unknown status, *N/A* not applicable, *NE* not evaluable, *ORR* objective response rate, *PD* progressive disease, *PD-L1* programmed death-ligand 1, *PR* partial response, *RECIST* Response Evaluation Criteria in Solid Tumors, *SD* stable disease, *UC* urothelial carcinoma.

**FIGURES**

**Fig. S1 Mean BGB-A333 serum concentration – time (+ SD) profiles in semi-log scale: (A) following single IV dose of BGB-A333 (Cycle 1, Phase 1a); (B) following multiple IV doses of BGB-A333 (Cycle 5, Phase 1a); (C) following single IV dose of 1350 mg BGB-A333 monotherapy (Phase 1a) or 1350 mg BGB-A333 + 200 mg tislelizumab combination therapy (Phase 1b); and (D) following multiple IV doses of 1350 mg Q3W BGB-A333 monotherapy (Phase 1a) or BGB-1350 mg Q3W A333 + 200 mg Q3W tislelizumab combination therapy (Phase 1b) (PK analysis set)**


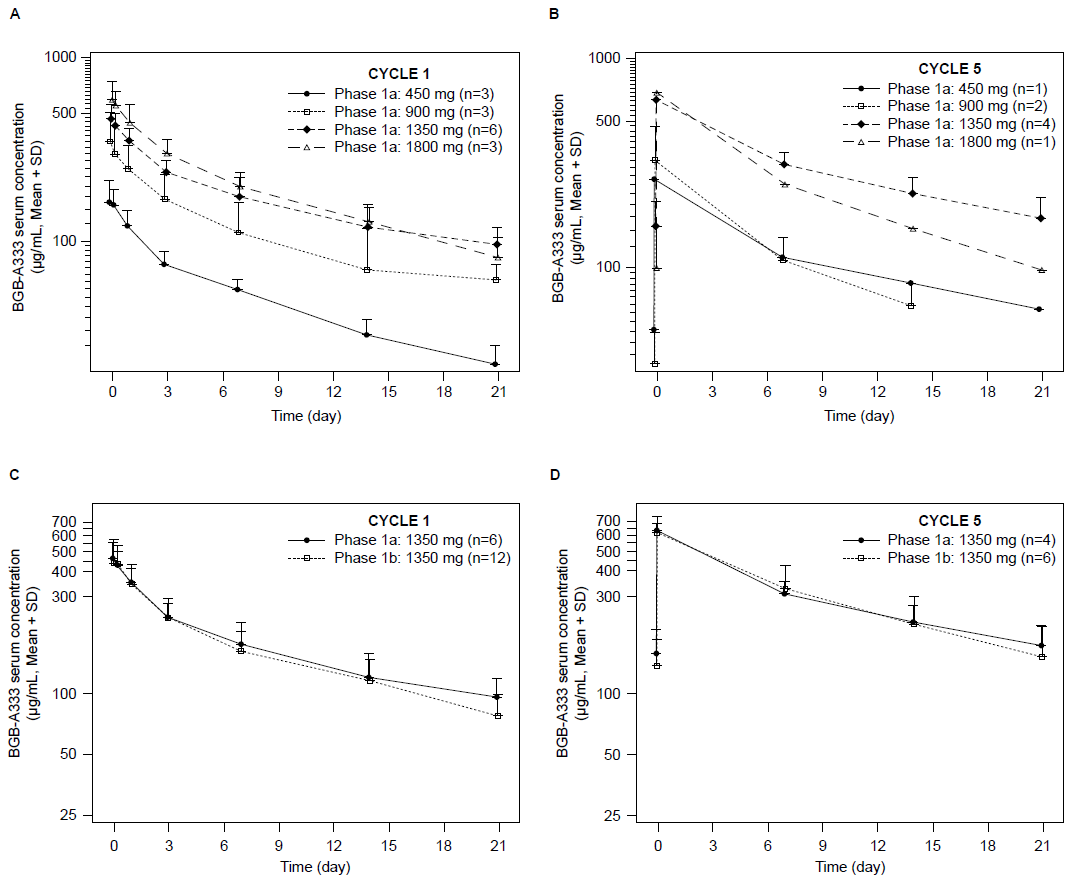


*IV* intravenous, *PK* pharmacokinetic, *SD* standard deviation.

**Fig. S2 (A) Kaplan-Meier plot of progression-free survival (Phase 1b; intent-to-treat set); (B) Kaplan-Meier plot of progression-free survival (Phase 2; intent-to-treat set)**


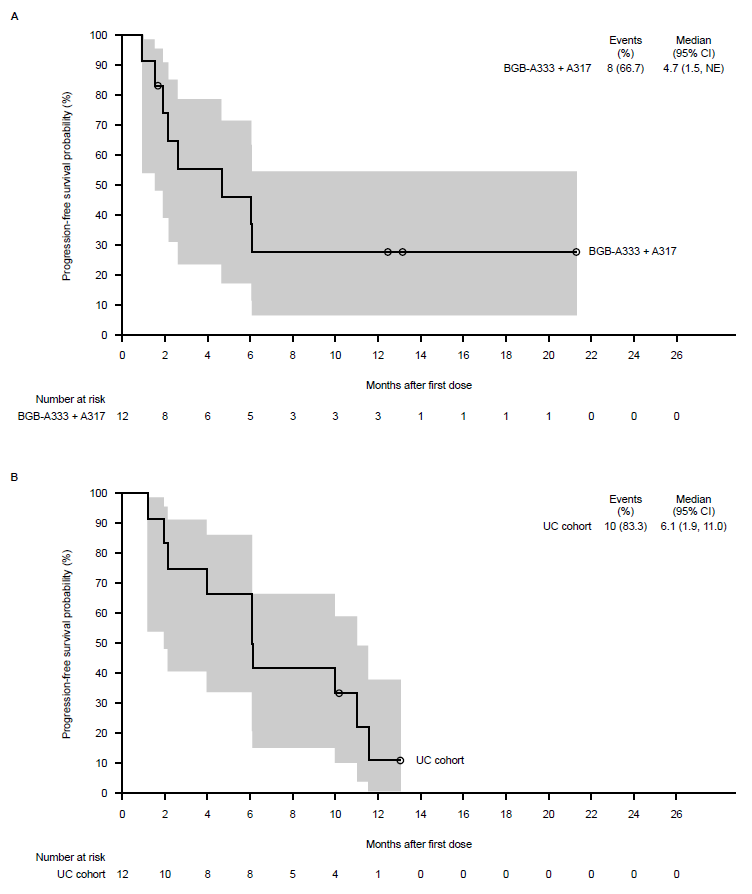


Concentration values equal to 0 were not included in the semi-log scale figure.

*CI* confidence interval, *NE* not estimable, *UC* urothelial cancer.

**Fig. S3 Study flow, (A) Phase 1a; (B) Phase 1b and Phase 2**


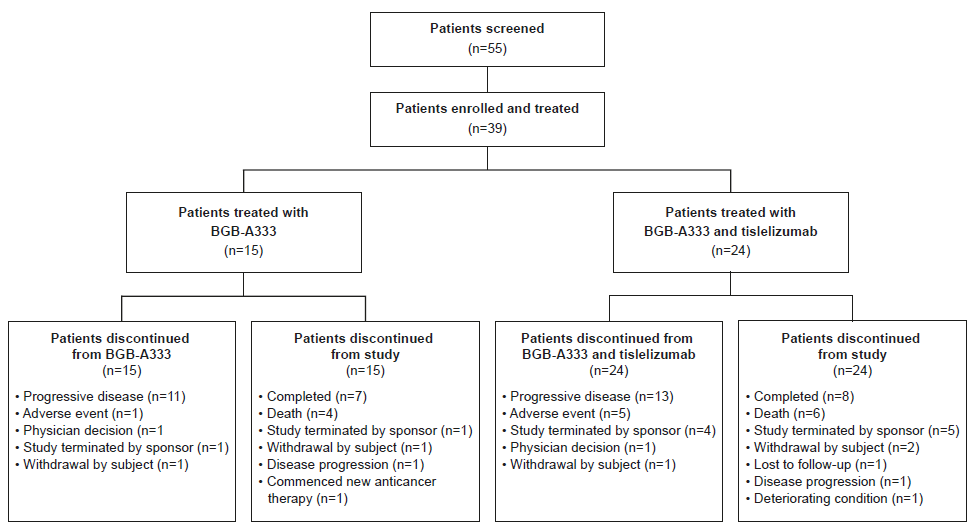

Supplement: Supplementary file 1 — SUPPLEMENTARY INFORMATION [file 41416_2022_2128_MOESM1_ESM.docx]
